# Deubiquitinating enzyme USP10 promotes osteosarcoma metastasis and epithelial–mesenchymal transition by stabilizing YAP1

**DOI:** 10.1002/cam4.6074

**Published:** 2023-05-15

**Authors:** Jianyong Deng, Xuan Yi, Zuxi Feng, Jie Peng, Dan Li, Chen Li, Binbin Deng, Shuaigang Liu, Souradeep Sahu, Liang Hao

**Affiliations:** ^1^ Department of Orthopedics Second Affiliated Hospital of Nanchang University Nanchang China; ^2^ Second Affiliated Hospital of Nanchang University Nanchang China; ^3^ Department of Oncology Second Affiliated Hospital of Nanchang University Nanchang China

**Keywords:** deubiquitination, EMT, osteosarcoma, USP10, YAP1

## Abstract

**Background:**

Osteosarcoma (OS) is a fatal adolescent tumor, which is susceptible to remote metastases at an early stage, and its treatment remains a major challenge. ubiquitin‐specific protease 10 (USP10) is primarily located in the cytoplasm and can therefore deubiquitinate various cytoplasmic proteins. However, the expression and mechanism of USP10 in OS remain ambiguous. The aim of this study was to explore how USP10 affects Yes‐associated protein1 (YAP1) to influence the metastasis and epithelial–mesenchymal transition (EMT).

**Methods:**

Western blotting, qRT‐PCR, and immunohistochemical (IHC) analyses were performed to evaluate USP10 and YAP1 levels. Using wound healing and transwell tests, the roles and molecular pathways of USP10 and YAP1 ability to migrate and invade of OS were investigated, and cell morphological alterations were examined using phalloidin staining.

**Results:**

Our results indicated that USP10, a new type of deubiquitinating protease, is increased in OS tissues and cells contrasted with adjacent healthy tissues. Overexpression of USP10 correlated with tumor size, distant metastasis, and TNM stage, and was an independent factor of poor prognosis in OS patients. Also, USP10 expression is closely connected with the incident of OS metastasis and tumor size. Functional assays revealed that USP10 knockdown suppressed cell migrating and invading ability and inhibited the EMT of OS cells in vivo and in vitro. In addition, we showed that USP10 knockdown decreased the levels of YAP1, which is an important positive regulator of migration and invasion in many cancers. We also found a significant positive correlation between USP10 and YAP1 levels, further demonstrating that USP10‐induced migration and EMT are based on YAP1 in OS cells. In a mechanistic way, USP10 stabilizes the expression of YAP1 by mediating its deubiquitination in OS cells.

**Conclusion:**

Together, this study showed that USP10 can directly interact with YAP1 to reduce ubiquitinated YAP1, thereby stabilizing its protein levels and affecting EMT and distant metastasis in OS cells.

## 1 INTRODUCTION

Osteosarcoma (OS) is considered of the highest prevalent primary bone cancers in adolescents and children and is highly malignant.^1^ In recent decades, with the rapid development of medicine, the 5‐year overall survival rate of individuals with early OS after surgical resection and adjuvant chemotherapy has reached as high as 50%–60%.^2^, ^3^, ^4^ However, OS has the characteristics of early lung metastasis^5^ and a high recurrence rate,^6^ and recurrence mostly manifests as distant lung metastasis. It has been reported that the prognosis of metastatic patients remains poor after treatment,^7^ and the overall survival rate is often less than 20%.^8^, ^9^ Consequently, there is an immediate necessity to locate effective molecular targets to reduce distant metastasis in OS and improve the 5‐year survival rate of patients. Consequently, further clarification of the pathogenesis of OS distant metastasis will establish a new conceptual framework for OS‐tailored treatment.

Ubiquitin‐specific protease 10 (USP10) is one of the main members of the family of deubiquitinating enzymes.^10^ It is located in zone 1 of the long arm of chromosome 16 and contains 18 exons.^11^ USP10 is composed of 798 amino acids, with a relative molecular weight of approximately 92.9 kD, and the main molecular domains consist of Ataxin2C and a USP.^10^ Its molecular functional structures are mainly cysteine‐type endopeptidase and ubiquitin sulfhydryl esterase. USP10 is mainly located in the cytoplasm. It can deubiquitinate various proteins in the cytoplasm and regulate cell proliferation, the cell cycle, apoptosis, and autophagy through interaction with G3BP.^12^ Recent research has suggested that USP10 modulates cell proliferation, migration, and apoptosis. Studies have shown that USP10 is strongly expressed in various tumor tissues, including hepatic,^13^ breast,^14^ and prostate cancer.^12^, ^15^ USP10 can inhibit the proliferation and metastasis of lung cancer cells by upregulating PTEN expression.^16^ USP10 can also induce epithelial–mesenchymal transition (EMT) in prostate cancer via deubiquitination of substrate proteins.^15^ However, the expression and mechanism of USP10 in OS remain unclear.

EMT is the process of transformation from epithelial to mesenchymal cells.^17^ It confers the ability to metastasize and invade cells, which includes stem cell characteristics, reduced apoptosis, and aging, and promotes immunosuppression.^18^ It not only plays a key role in developmental processes but also participates in tissue healing, organ fibrosis, and cancer occurrence. EMT of tumor cells plays a key part in the distant metastasis of tumors. Throughout EMT, polarized epithelial cells undergo a variety of complex changes that transform into mesenchymal cells with high migratory and invasive capabilities. The typical epithelial cell marker is E‐cadherin, which mainly exists on the surface of epithelial cell membranes, whereas vimentin and N‐cadherin are mainly found in mesenchymal‐derived cells and are important markers of mesenchymal cells.^19^, ^20^

Numerous recent research has proven that the Hippo/YAP signaling system is a remarkably conserved growth controlling signaling pathway, which plays a vital part in tumor cell growth,^21^ and distant metastasis.^22^ As a crucial downstream effector of the Hippo signaling pathway, YAP1 has been shown to be overexpressed in most tumors.^23^ Overexpression of YAP1 can induce EMT in the triple‐negative breast cancer cell,^24^ and Helicobacter pylori CagA can promote EMT in stomach cancer cells by inducing the expression of YAP1.^25^ Our earlier research proved that overexpression of YAP1 in OS promotes OS cell proliferation.^26^ Nonetheless, YAP1function of the EMT stays unknown.

This research aimed to elucidate the roles of USP10 and YAP1 in osteosarcoma EMT as well as distant metastasis. Our study showed that USP10 expression is increased in OS and is correlated with disease progression and poor prognosis in OS patients. USP10 enhances EMT and distant metastasis of OS. We further investigated the mechanisms of USP10 and YAP1 in osteosarcoma EMT, confirming that USP10 regulates YAP1 expression through altering the breakdown and ubiquitination of the downstream gene YAP1, thereby affecting the EMT and distant metastasis of osteosarcoma. To sum up, this research indicates that USP10 is a potential therapeutic target for OS and provides a theoretical basis for targeted therapy.

## 2 MATERIALS AND METHODS

### 2.1 Patients and human tissue specimens

Following receiving written consent from the subjects and permission from the Ethics Committee of the Second Affiliated Hospital of Nanchang University, 51 samples of OS and surrounding tissues were obtained from the Second Affiliated Hospital and the First Affiliated Hospital of Nanchang University. All specimens were pathologically diagnosed with OS. All patients were followed up for 3 years. All specimens were kept at −80°C in a refrigerator and used for western blotting, qRT‐PCR, and immunohistochemical (IHC) analyses.

### 2.2 Cell lines

The human osteosarcoma (OS) cell lines MG‐63, 143B, Saos‐2, and U2‐OS as well as the human osteoblast cell line HfobI‐19, were acquired through the Chinese Academy of Sciences. The 143B cell line was cultivated in Dulbecco's modified Eagle's medium (DMEM, American Type Culture Collection); the MG‐63 cell line was grown in Minimum Essential Medium (MEM, PM150410, Procell Life Science& Technology); the Saos‐2 and U2‐OS cell lines were grown in McCoy's 5A (PM150710, Procell Life Science & Technology) media were enriched with 10% Fetal bovine serum (FBS; Gibcod), 1% penicillin, and 1% streptomycin. The OS cell lines were cultivated in an incubator at 37°C, with 5% CO_2_ and 95% humidity. The osteoblast cell line was grown in an incubator at 34°C, with 5% CO_2_ and 95% humidity. Cell homology, with all the above cell lines, was confirmed through a series of short tandem repeats. All cell lines were used within 6 months of being procured.

### 2.3 Immunohistochemical analysis

Fresh frozen OS tissue and adjacent tissue specimens were fixed using 4% paraformaldehyde, then immersed in paraffin, sectioned to the thickness of 3 μm, degreased, and hydrated. The tissues were incubated overnight with antibodies against USP10 (Abcam, ab109219, 1:100 dilution) and YAP1 (Abcam, ab52771, 1:200 dilution) at 4°C. The sections were then incubated with the corresponding secondary antibodies for 1 h at room temperature, followed by microscopic observation. The staining intensity and the percentage of positive cells were scored blindly, randomly, and semi‐quantitatively. The total staining index was calculated by the score and score multiplied by 0–9 points, and the final score was USP10 non‐overexpression (0–1) or USP10 overexpression (2–9).

### 2.4 Wound healing assay

OS cells in the logarithmic growth stage were transfected with shRNAs for 4–6 h, new complete media was supplied. After 2 days, the cells were planted in a 6‐well plate at a concentration of 1 × 10^6^ cells for each well, using a complete culture medium. After the cells had grown to cover the 6‐well plate, a 200‐μL sterile pipette point was utilized to scrape off a bare area of the same width and wash off the cell debris with PBS. The cells were cultured in an incomplete medium, placed in an incubator for 24 h, and then observed using a microscope to take pictures of the wound healing.

### 2.5 CCK8 assays and EdU assay

CCK8 assays and EdU assay were performed as previously described.^27^

### 2.6 Transwell assay

OS cells in the logarithmic growth phase were mixed in 200 μL serum‐free media and seeded into the upper chamber of the transwell. Following the addition of 60 μL of serum‐containing complete media to the lower chamber, the cells were placed into the incubator. Following being incubated for 24 h, the nonpenetrative cells on the inner side of the upper lumen membrane were removed using a swab. The cells adhered to the bottom of the membrane were fixed with 4% paraformaldehyde for 30 min and then dyed using 0.1% crystal violet solution for 30 min. The cells were washed with PBS solution three times in minutes, observed randomly under a microscope, and for counting.

### 2.7 Immunofluorescence

At a concentration of (1 × 10^4^), OS cells that were in the logarithmic growth phase were seeded into a petri plate, cultivated for 24 h, washed with pre‐cooled PBS, fixed with paraformaldehyde at a concentration of 4%, allowed to rest at room temperature for 15 min, and then washed again. After permeabilizing the cells using Triton X‐100 (0.1%) for 30 min, the cells were bound by BSA at room temperature for 1 h. Following an overnight incubation at 4°C. with diluted primary antibodies against YAP, E‐cadherin, and N‐cadherin and vimentin, the cells were then treated with Alex‐Fluor‐568‐ or FITC‐conjugated anti‐mouse secondary antibodies. The cells were observed under a laser scanning confocal microscope.

### 2.8 Quantitative reverse transcription‐PCR (qRT‐PCR), western blotting, and co‐immunoprecipitation (Co‐IP) analyses

qRT‐PCR, western blot, and co‐IP assays were conducted according to the earlier description.^28^ The primers used for qRT‐PCR were: USP10: forward: 5‐AATAAAGGGAACTGGTGC‐3, reverse: 5‐CTATCATGGGTGTTGACGT‐3; YAP1: forward: 5‐GCAACTCCAACCAGCAGCAACA‐3, reverse: 5‐CGCAGCCTCTCCTTCTCCATCTG‐3; and GAPDH: forward: 5‐AGCCTCAAGATCATCAGCAATG‐3, reverse: 5‐CCATCACGCCACAGTTTCC‐3. The antibodies used for western blotting were: USP10 (Abcam, ab109219, 1:1500 dilution), YAP1 (Abcam, ab52771, 1:1000 dilution), vimentin (Abcam, ab92547, 1:1500 dilution), E‐cadherin (Abcam, ab40772, 1:1500 dilution), N‐cadherin (Abcam, ab18203, 1:1500 dilution), and GAPDH (Abcam, ab9485, 1:5000 dilution).

### 2.9 Lentivirus construction, recombinant plasmid, and siRNA transfection

The USP10 shRNA, shNC, USP10 overexpression, and control overexpression lentiviruses were purchased from GenePharma. qRT‐PCR and western blotting confirmed the stable knockdown and upregulation of USP10. Plasmids overexpressing YAP1 and YAP1‐siRNA were obtained from GenePharma. Using the Lipofectamine 3000 Transfection Reagent, the plasmids were transfected into the OS cells in a manner that was compliant with the instructions provided by the supplier (Invitrogen).

### 2.10 Phalloidin staining

OS cells in the logarithmic growth phase were inoculated in a confocal culture dish, cultured in a serum‐free medium for 1 day, fixed on ice with 3.75% paraformaldehyde for a period of 15 min, rinsed thrice using PBS; after that, they were washed by 0.1% Triton X‐100. They were then permeabilized with PBS solution at room temperature for 15 min, rinsed once again using PBS three times, and incubated with phalloidin conjugated to YF@488 (UE Everbright@ Inc) for 20 min in the dark. A laser scanning microscope (Leica) was used to observe the images.

### 2.11 Growth and metastasis assay in vivo

In vivo proliferation and metastasis experiments, osteosarcoma cells (5 × 10^6^ in 100 mL of PBS) were inoculated on the back of 5‐week‐old nude mice (female BALB/c‐nu/nu) and injected through the tail vein, and the nude mice were sacrificed 30 days later for collection tumor and lung tissue. Tumors were weighed, photographed, and stained with HE.

### 2.12 Statistical analysis

The tests were carried out more than three times, and the collected statistics are shown as the mean ± standard deviation (SD). For conducting the statistical analysis, GraphPad Prism 8 and SPSS 26.0 statistical tools were utilized. A Student's *t*‐test with no matching pair of data was used to analyze the variations between the two sets of information. When analyzing the data from various groups, a one‐way analysis of variance (ANOVA) was carried out. At a level of statistical significance <0.05, variations were regarded as significant.

## 3 RESULTS

### 3.1 USP10 expression is upregulated in OS and significantly correlated with the prognosis of OS patients

**FIGURE 1:**
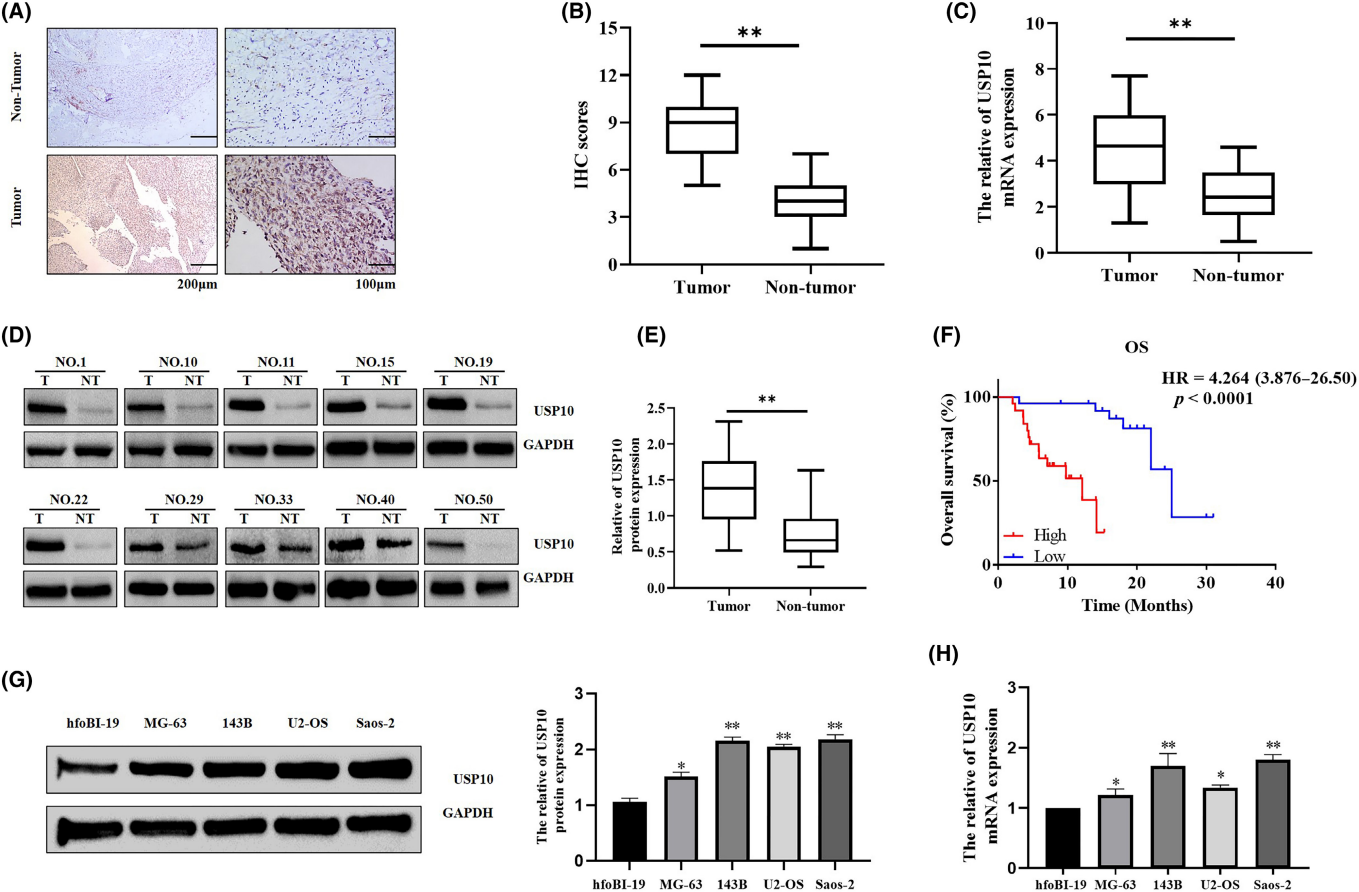
Expression of relative USP10 mRNA and protein levels in osteosarcoma tissues and cell lines. (A, B) Representative photos (A) and quantification (B) of USP10 IHC dying in 51 paired OS tumor and their corresponding adjacent tissues (**p < 0.01, N = normal, T = tumor). (C) Detection of USP10 mRNA levels in OS and paired non‐tumorous tissues by qRT‐PCR (*n* = 51, **p < 0.01). (D, E) Western blotting analyses of USP10 protein levels in OS tissues and paired non‐tumor tissues (*n* = 51, **p < 0.01). (F) Kaplan–Meier analysis of the correlation between the USP10 level and overall survival of OS patients with high and low USP10 expression. (***p < 0.001). (G, H) Western blotting and qRT‐PCR analyses of USP10 protein and mRNA levels in OS cells (Saos‐2, U2‐OS, MG‐63, and 143 B) and hfoBI‐19 cells (*p < 0.05, **p < 0.01).

IHC analysis of 51 cases of OS and its adjacent tissues indicated that USP10 expression was increased in 68.63% (35/51) of OS tissues but was only detected in 17.65% (9/51) (Figure 1A,B) of adjacent tissues. Tumor and adjacent tissues were subjected to qRT‐PCR and western blotting, and the findings confirmed that USP10 was highly expressed in OS tissues (Figure 1C–E). Furthermore, we investigated the relationship between USP10 levels and different clinicopathological features of OS and found that higher USP10 expression was correlated with larger tumor size, more metastases and worse TNM stage (Table 1). Similarly, we found that USP10 levels were associated with prognosis in OS patients. Survival analysis using the Kaplan–Meier method showed that patients with high USP10 expression had worse overall survival than those with low USP10 expression (Figure 1F). Additionally, univariate and multivariate logistic regression analyses indicated that USP10 was an independent predictor of poor prognosis in OS patients (Table 2). At the same time, our results also showed that USP10 expression in the OS cell line was significantly increased compared with that in typical osteoblasts (Figure 1G,H **)**. Taken together, the above results indicated that USP10 was significantly overexpression in OS, and the increase of USP10 was correlated with disease progression and poor prognosis in OS patients.

**TABLE 1:** Association of USP10 expression with clinical features in osteosarcoma patients.

Classification	Total	USP10 expression		χ^2^	*p*‐value^a^
		High	Low		
Age				0.155	0.877
<18	28	14	14		
≥18	23	11	12		
Gender				0.444	0.657
Female	29	15	14		
Male	22	10	12		
Tumor size (cm)				2.241	**0.025** *
<5	18	5	13		
≥5	33	20	13		
Location				1.552	0.121
Upper limb bone	24	9	15		
Lower limb bone	27	16	11		
TNM stage				1.980	**0.048** *
I/II	26	9	17		
III/IV	25	16	9		
Distant metastasis				4.561	**0.033** *
M0	32	12	20		
M1	19	13	6		
Vital status				1.570	0.210
Alive	29	12	17		
Dead	22	13	9		
Recurrence				0.954	0.329
Absence	32	14	18		
Presence	19	11	8		

Bold values emphasize that the *p*‐value is statistically significant.

^a^ Chi‐squared test.

* *p* < 0.05.

**TABLE 2:** Univariate and multivariate analyses of the prognostic factors in OS patients using a Cox regression model.

Parameters	Univariate analysis			Multivariate analysis		
	HR	95% CI	*p*‐value	HR	95% CI	*p*‐value
Age (<18 vs. ≥18)	1.50	0.64–3.51	0.349	―	―	―
Gender (female vs. male)	2.35	0.91–6.03	0.076	―	―	―
Tumor size (<5 vs. ≥5)	5.81	1.70–19.83	**0.005** **	2.11	0.47–9.44	0.327
Location (upper limb vs. lower limb)	1.50	0.65–3.48	0.345	―	―	―
Stage (I/II vs. III/IV)	9.25	2.15–39.85	**0.003** **	2.69	0.56–13.03	0.219
Distant metastasis (M0 vs. M1)	3.01	1.29–7.04	**0.011** *	1.94	0.49–7.79	0.348
Recurrence (absence vs. presence)	1.21	0.46–3.18	0.698	1.35	0.50–3.62	0.551
USP10 expression (high vs. low)	1.11	1.05–1.18	**3.64E‐04** ***	1.11	1.03–1.20	**0.005** **

Bold values emphasize that the *p*‐value is statistically significant.

Abbreviations: CI, confidence interval; HR, hazard ratio.

* *p* < 0.05

** *p* < 0.01

*** *p* < 0.001.

### 3.2 USP10 affects the invasion and migration of OS cells

**FIGURE 2:**
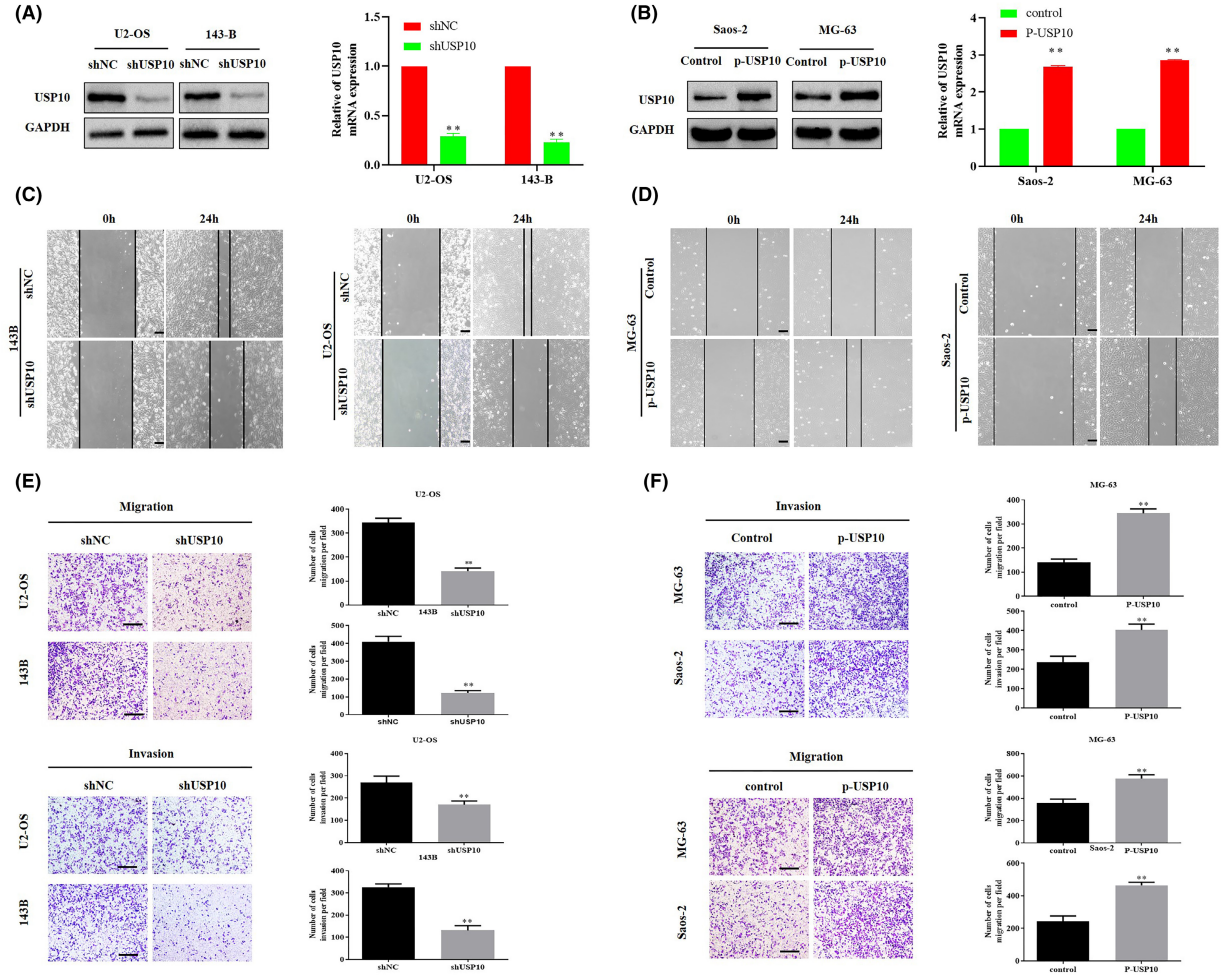
USP10 affects the invasion and migration of OS cells. (A) Western blotting and qRT‐PCR experiments of USP10 protein and mRNA levels in U2‐OS and 143‐B cell lines transfected with shNC and shUSP10 plasmids. (B) P‐USP10 plasmid was used to transfect Saos‐2 and MG‐63 OS cell lines, western blot and qRT‐PCR confirmed USP10 rise. (C, E) Wound healing and transwell tests were utilized to identify the impact of knockdown USP10 in the migration and invasiveness of U2‐OS and 143‐B (**p < 0.01; magnification, 200×, scale bar, 100 μm). (D, F). Migration and invasion of Saos‐2 and MG‐63 cells transfected with control and p‐USP10 were studied using wound healing and transwell assays (**p < 0.01, magnification, 200×, scale bar, 100 μm).

For additional examination of the role of USP10 in OS cells, we transfected shUSP10, shNC, p‐USP10, and vector plasmids into OS cells in compliance with the supplier's guidelines, and we verified the effectiveness of the transfection using western blotting and qRT‐PCR (Figure 2A,B). Furthermore, we performed wound healing and transwell tests to confirm the OS cells' migrating and invading ability after USP10 knockdown and overexpression. The results indicated that USP10 downregulation in U2‐OS and 143B cells reduced their migration and invasiveness abilities (Figure 2C,E). Simultaneously, upregulation of USP10 in MG‐63 and Saos‐2 cells increased their migration and invasiveness abilities (Figure 2D,F). To sum up, the current results suggest that USP10 promotes the migration and invasiveness of OS cells.

### 3.3 Downregulation of USP10 can inhibit EMT in OS cells

**FIGURE 3:**
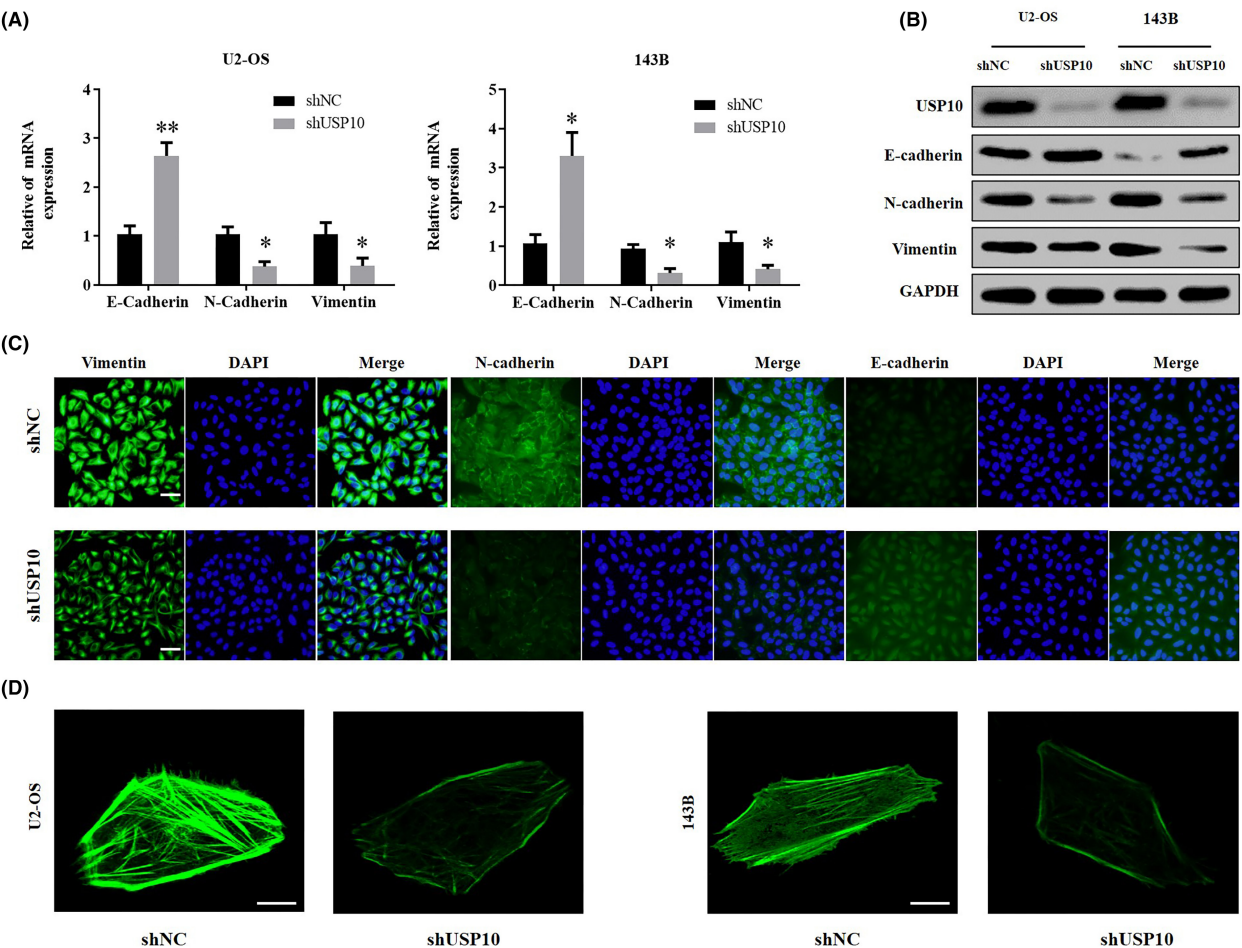
Downregulation of USP10 can inhibit EMT in OS cells. (A, B) qRT‐PCR and western blotting of EMT‐related proteins and their mRNA levels in shUSP10‐transfected cells compared to those observed in shNC‐transfected cells. (C) Immunofluorescence images depicting the influence of shUSP10 on vimentin, E‐cadherin, and N‐cadherin levels (blue: DAPI, scale bar: 20 μm). (D) Morphological alterations observed by phalloidin staining of actin in OS cells.

Our results confirmed EMT's active contribution in the invasiveness as well as migration of tumor cells. In this study, qRT‐PCR and western blot tests demonstrated that upon reduction of USP10 in U2‐OS and 143B cells, the mRNA and protein levels of EMT‐related genes N‐cadherin and vimentin reduced, while that of E‐cadherin increased (Figure 3A,B). In addition, we observed an abnormal expression of E‐cadherin, N‐cadherin, and vimentin using immunofluorescence (Figure 3C). Confocal imaging was used to observe the morphological changes in cells stained with phalloidin (Figure 3D). In brief, the above experiments confirmed that USP10 downregulation could inhibit EMT in OS cells.

### 3.4 TGF‐β can block the inhibitory effect of shUSP10 on EMT in OS cells

**FIGURE 4:**
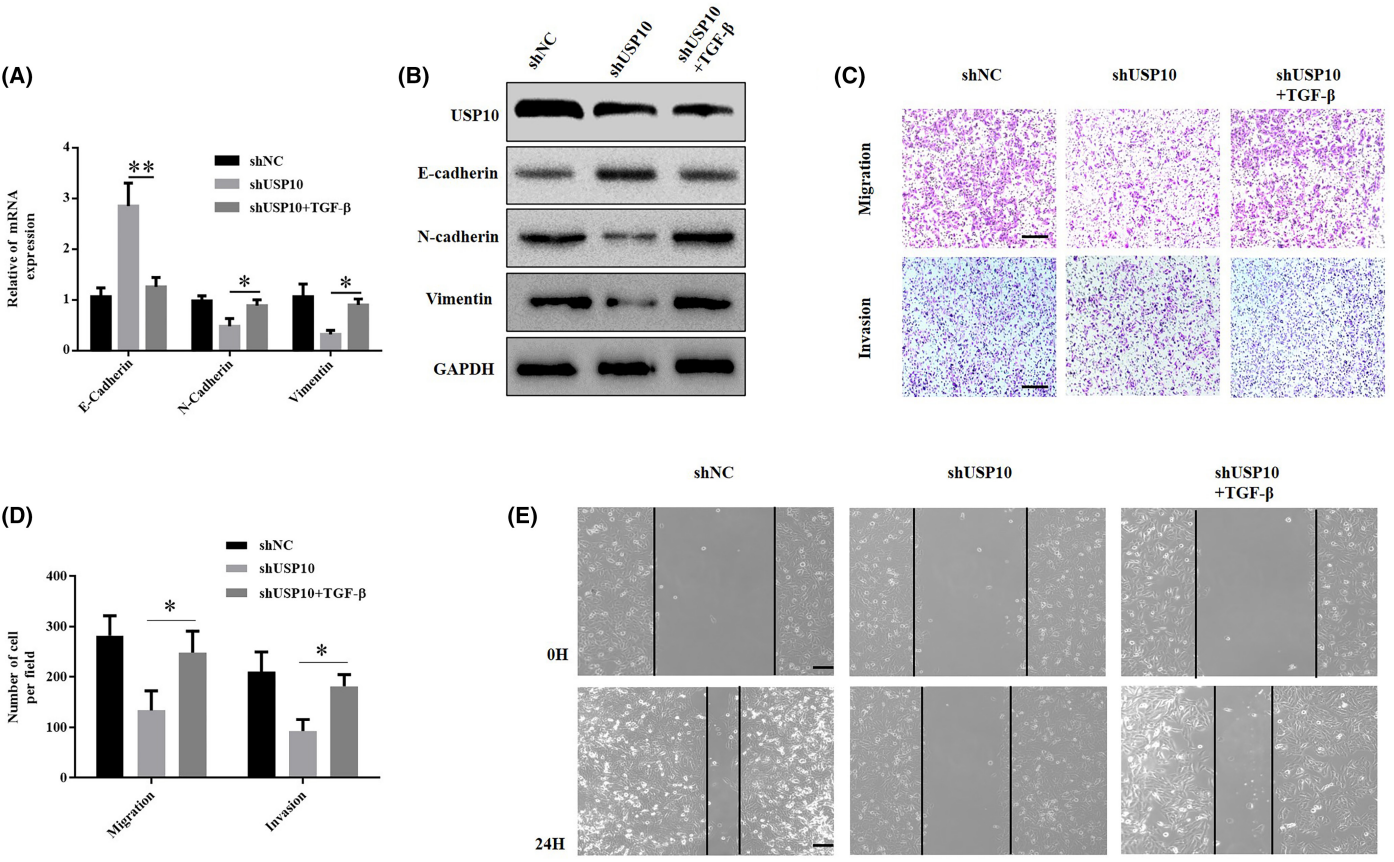
TGF‐β can block the inhibitory effect of shUSP10 on OS cell EMT. (A, B) The relative expression levels of EMT‐related mRNAs and proteins were identified by qRT‐PCR and western blotting after knockdown of USP10 in osteosarcoma cells and treatment with TGF‐β. (C–E) In osteosarcoma cells stably transfected with shNC/shUSP10 treated with TGF‐β, changes in migration and invasiveness capabilities were analyzed by transwell and wound healing tests (**p < 0.01, magnification, 200×, scale bar, 100 μm).

Transforming growth factor‐β (TGF‐β) performs a key part in EMT in various malignant tumors.^29^ Thence, we further verified that USP10 can regulate the EMT of OS cells by using TGF‐β. The OS cells transfected with shUSP10 were treated with TGF‐β (5 ng/mL). After 48 h, the mRNA as well as protein levels of N‐cadherin and vimentin elevated when contrasted with the control group, whereas those of E‐cadherin reduced (Figure 4A,B). Moreover, transwell and wound healing experiments revealed that relative to the control group, the migrating and invading capabilities of OS cells transfected with shUSP10 increased after the addition of TGF‐β (5 ng/mL) for 48 h (Figures 4C–E). In summary, these results demonstrated that TGF‐β could reverse the suppressing impact of shUSP10 on EMT in OS cells.

### 3.5 USP10 positively regulates YAP1 protein expression in osteosarcoma cells

**FIGURE 5:**
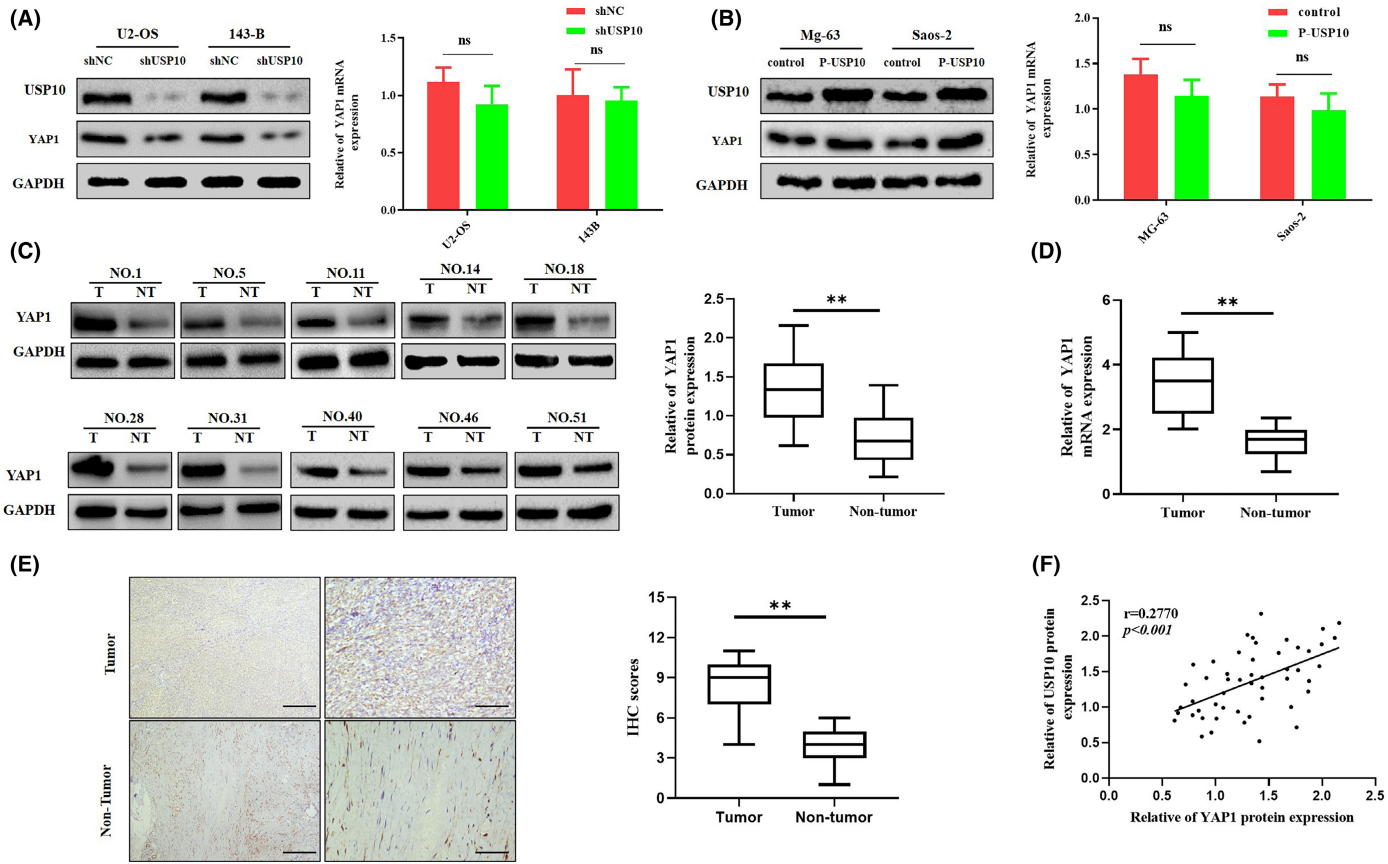
USP10 positively regulates YAP1 protein expression in osteosarcoma cells. (A, B) In response to the knockdown or upregulation of USP10 expression levels in OS cells, the protein expression levels of YAP1 also changed significantly, however, the change in its mRNA levels was not statistically significant (ns, not significant). (C, D) Detection of YAP1 protein and mRNA expression in OS and paired non‐tumor tissues via western blot and qRT‐PCR. (**p < 0.01; N, normal; t, tumor). (E) Representative images (left) and quantification (right) of YAP1 IHC staining in 51 paired OS tumor and their corresponding adjacent tissues (**p < 0.01). (F) The scatter plot depicted that the protein expression levels of YAP1 and USP10 in OS tissues were positively correlated.

We have previously confirmed that the Hippo/YAP signaling pathway performs key part in the development of OS. YAP1 is overexpressed in OS cells and promotes their growth.^26^ In this study, USP10 downregulation in OS cells was accompanied by a decrease in YAP1 protein levels. Conversely, when USP10 was overexpressed, YAP1 protein levels increased, but its mRNA levels remained unchanged (Figure 5A,B). At the same time, qRT‐PCR, western blotting, and IHC analyses proved that YAP1 expression increased in OS tissues more than in adjacent tissues (Figure 5C–E). In addition, a scatter plot assessment demonstrated that the protein expression patterns of USP10 and YAP1 were positively correlated (Figure 5F). In summary, USP10 positively regulates YAP1 protein expression in osteosarcoma cells.

### 3.6 YAP1 is essential for USP10‐mediated EMT and migration of OS cells

**FIGURE 6:**
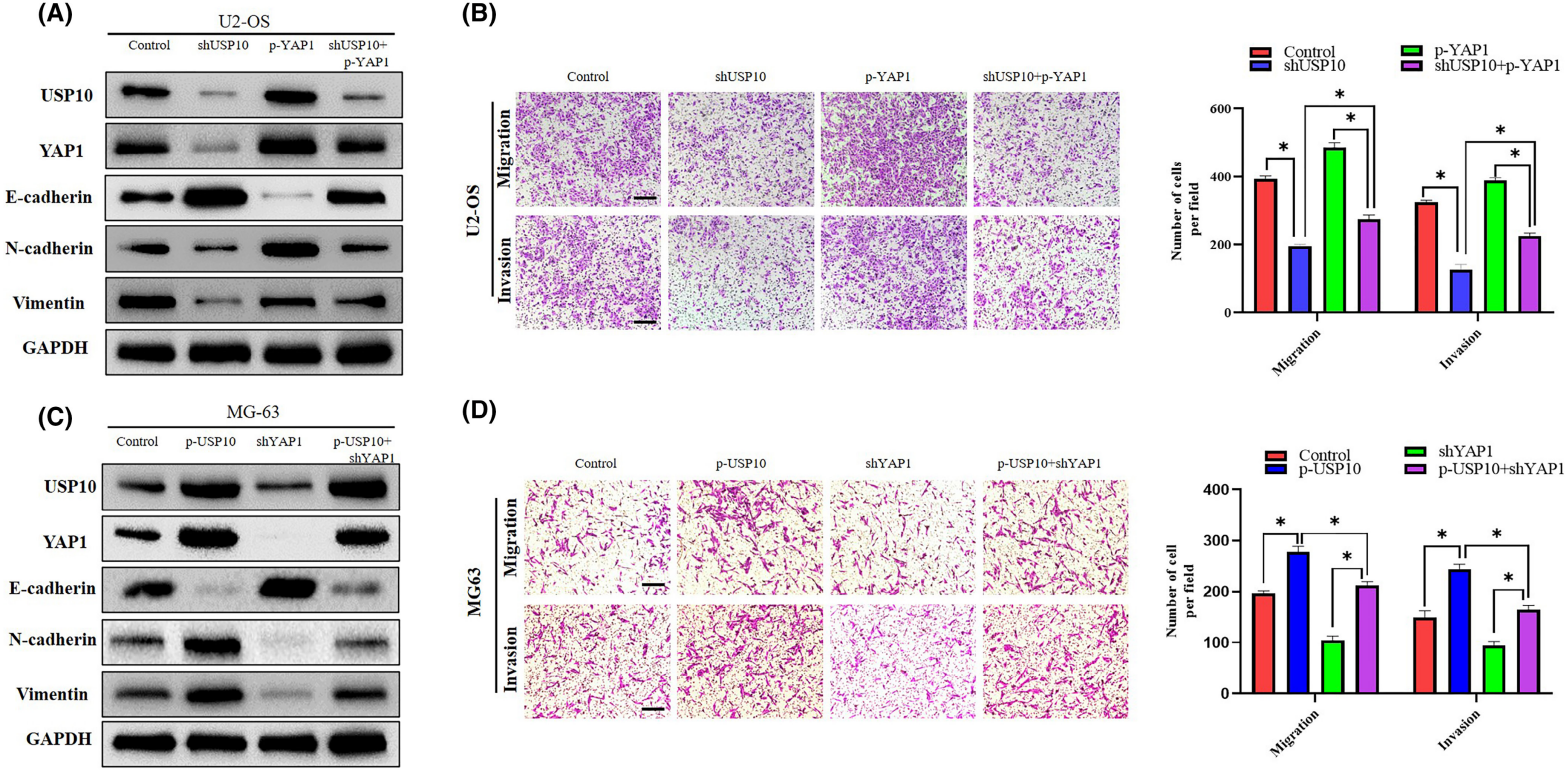
YAP1 is essential in USP10‐mediated EMT and migration of OS cells. (A) The P‐YAP1 plasmid was transfected into U2‐OS cells stably transfected with shUSP10, and the expression of YAP1 and EMT‐related proteins was shown by western blotting. (B) In U2‐OS cells, the decrease in invasion and migration induced by shUSP10 could be partially offset by P‐YAP1 as detected by transwell test (*p < 0.05, **p < 0.01). (C) The shYAP1 plasmid was transfected into MG‐63 cells stably transfected with P‐USP10, and the expression of YAP1 and EMT‐related proteins was shown by western blotting. (D) In MG‐63 cells, the enhancement of invasion and migration mediated by P‐USP10 was partially counteracted by shYAP1, as determined by transwell assays (*p < 0.05, **p < 0.01).

As mentioned above, we discovered that YAP1 plays a role downstream of USP10. Therefore, we investigated the function of YAP1 in the EMT of osteosarcoma cells. First, we upregulated YAP1 in OS cells that had been transfected with the shUSP10 plasmid and confirmed its overexpression using western blotting. Meanwhile, the protein levels of vimentin and N‐cadherin elevated, whereas that of E‐cadherin reduced (Figure 6A). The findings of the Transwell experiment revealed that shUSP10 inhibited the invasiveness and migration of OS cells. Moreover, the introduction of the YAP1 overexpression plasmid reversed this inhibitory effect (Figure 6B).

Next, we knocked down YAP1 in OS cells transfected with the p‐USP10 plasmid and demonstrated its silencing effect by western blotting. Meanwhile, the protein levels of N‐cadherin and vimentin decreased, whereas that of E‐cadherin increased (Figure 6C). The results of the transwell assay showed that p‐USP10 promoted the invasive and migratory abilities of OS cells. However, the introduction of a plasmid that silences YAP1 inhibited this promoting effect (Figure 6D). Taken together, these results indicate that YAP1 mediates EMT and migration induced by USP10 in OS cells.

### 3.7 Downregulation of USP10 can inhibit the growth of OS cells in vitro, inhibit the growth and metastasis in vivo

**FIGURE 7:**
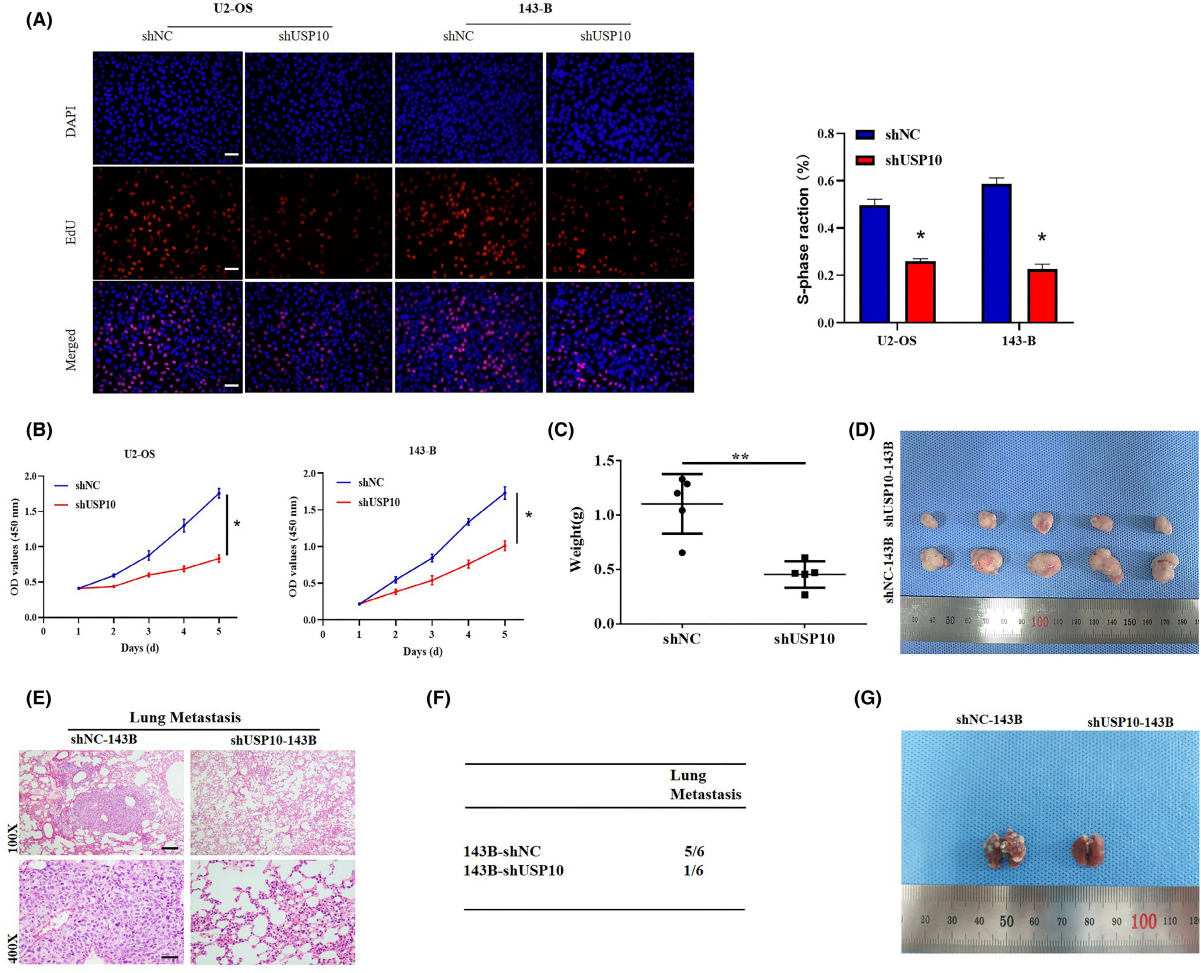
Downregulation of USP10 can inhibit the growth of OS cells in vitro, inhibit the growth and metastasis in vivo. (A, B) EdU and CCK8 assays showed that shUSP10 cell proliferation was inhibited (*p < 0.05). (C, D) Nude mice were subcutaneously inoculated with osteosarcoma cells (143B) transfected with shUSP10 and shNC plasmids to observe tumor formation in vivo, and tumors were collected and weighed 30 days later (**p < 0.01). (E) Osteosarcoma cells (143B) were transfected with shUSP10 and shNC plasmids into the tail vein of nude mice, and lung tissues were collected for HE staining 30 days later. (F) Prevalence of lung metastases in shNC and shUSP10 groups of nude mice. (G) Representative photographs of the lungs in shNC and shUSP10 mice.

As shown in Figure 7A, EdU assays showed that USP10 downregulation inhibited OS cell growth. In addition, CCK8 assays confirmed that USP10 knockdown inhibited the proliferation of U2‐OS and 143B cells (Figure 7B). For examining the impacts of USP10 on OS in vivo, we constructed a tumor xenotransplantation model, in which the shUSP10 OS cells were subcutaneously inoculated and administrated into the tail vein of nude mice, respectively. Nude mice were executed after 30 days of growth. Tumor and lung tissues were collected for weighing and photographing, and the lung tissue was stained with HE. Histological analysis showed that lung metastasis occurred in five cases in the shNC group. The above data indicated that xenografts derived from the shUSP10 group grew at a significantly slower rate in 143B cells than those derived from the control group (Figure 7C,D). And then, only one case of lung metastasis was observed in the shUSP10 group (Figure 7E,F). Representative photographs of the lungs from the shNC and shUSP10 groups are shown in Figure 7G. Collectively, these results show that downregulation of USP10 can inhibit the proliferation and metastasis of OS cells in vivo.

### 3.8 USP10 stabilizes the expression of YAP1 by mediating its deubiquitination in OS cells

**FIGURE 8:**
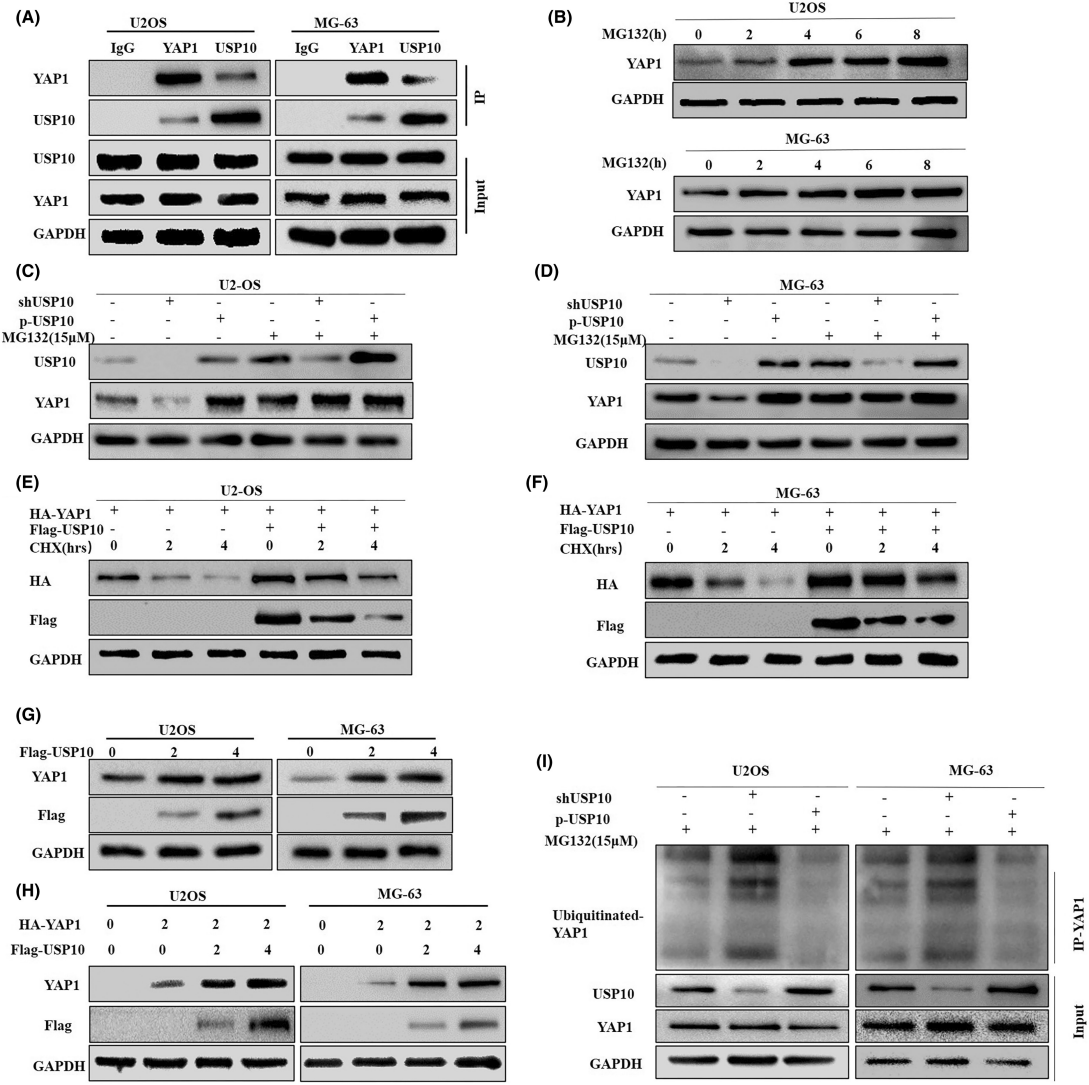
USP10 stabilizes the expression of YAP1 by mediating its deubiquitination in OS cells. (A) The co‐IP results confirmed that USP10 and YAP1 interact directly in osteosarcoma cells. (B) The expression of YAP1 in U2OS and MG‐63 osteosarcoma cells treated with protease inhibitor MG132 (15 μM) was detected by western blotting. (C, D) Western blot results showed that the expression level of YAP1 did not change significantly after knockdown or upregulation of USP10 osteosarcoma cells treated with MG132. (E, F) Osteosarcoma cells U2‐OS and MG‐63 were transfected with the plasmid encoding HA‐YAP1, in the presence or absence of the Flag‐USP10 plasmid. It was then treated with CHX (20 μM), and finally, the interpretation of YAP1 was observed by western blotting. (G) Endogenous YAP1 expression levels were measured by transfecting OS cells with different doses of Flag‐USP10 plasmid, (H) U2‐OS and MG‐63 cells were transfected by plasmid encoding HA‐YAP1 or in combination with Flag‐USP10 plasmid. YAP1 expression was observed by an anti‐HA antibody. (I) Osteosarcoma cells were transfected with knockout/overexpressing USP10 plasmids and treated with MG132 (15 μM), and ubiquitinated YAP1 levels were assessed by western blotting with anti‐Ub antibody.

To elucidate USP10 mechanism for regulating YAP1, we confirmed that USP10 can directly bind to YAP1 by Co‐IP (Figure 8A). In our previous study, we showed that YAP1 can be degraded by ubiquitination in OS cells.^26^ The results of a previous study confirmed that USP10 can stabilize substrate proteins by inhibiting their ubiquitination‐mediated degradation through deubiquitination.^30^ Therefore, we speculated that USP10 could stabilize YAP1 protein expression by inhibiting ubiquitination and degradation of YAP1. In eukaryotic cells, the ubiquitin–proteasome pathway is the major mechanism for the targeted degradation of proteins with short half‐lives, proteasome inhibitor (carbobenzoxy‐leu‐leu‐leucinal) can inhibit the degradation of proteins by ubiquitin.^31^ The experimental conditions of MG132 have been discussed in our previous experimental studies.^26^, ^28^ Therefore, we tested this hypothesis by treating OS cells with MG132 (15 μM) for a specific time period. The results showed that with the accumulation of MG132 treatment time, the accumulation of endogenous YAP1 protein in U2OS and MG‐63 cells were more significant (Figure 8B). These outcomes agree with the earlier studies' findings.^26^

Next, to further study whether USP10 affects YAP1 protein degradation, we knockdown or overexpression of USP10 in osteosarcoma cells and examined the impact of various expression patterns of USP10 on YAP1 protein levels in cells treated with or without MG132. The findings showed that OS cells treated with MG132 show no changes in the expression of YAP1 protein due to the overexpression or silencing of USP10 (Figure 8C,D). Cycloheximide (CHX), it's a protein translation inhibitor. The experimental conditions of CHX have been discussed in our previous experimental studies.^26^, ^28^ Furthermore, protein degradation kinetic tests revealed a significant extension of the half‐life of YAP1 protein in U2‐OS and MG63 cells with upregulated USP10 (Figure 8E,F). These results confirm that USP10 affects YAP1 protein degradation.

We assessed the function of USP10 in YAP1 degradation. The findings showed that ectopic USP10 expression in a dose‐dependent manner led to a significant rise in endogenous YAP1 protein levels in U2‐OS and MG‐63 cells (Figure 8G). Furthermore, HA‐YAP1 and Flag‐USP10 were cotransfected into OS cells. USP10 upregulation was discovered to have a dose‐dependent impact on YAP1 expression, suggesting that USP10 can stabilize YAP1 expression (Figure 8H).

Finally, we examined the method through which USP10 stabilizes the expression of YAP1 protein; we treated shUSP10/P‐USP10 U2OS and shUSP0/P‐USP10 MG‐63 cells with MG132. Co‐IP outcomes demonstrated that downregulation of USP10 rose the ubiquitination of YAP1, and conversely, upregulation of USP10 decreased the ubiquitination of YAP1 (Figure 8I). In conclusion, the above results confirm that USP10 regulates the ubiquitination and breakdown of YAP1 to maintain its levels.

**FIGURE 9:**
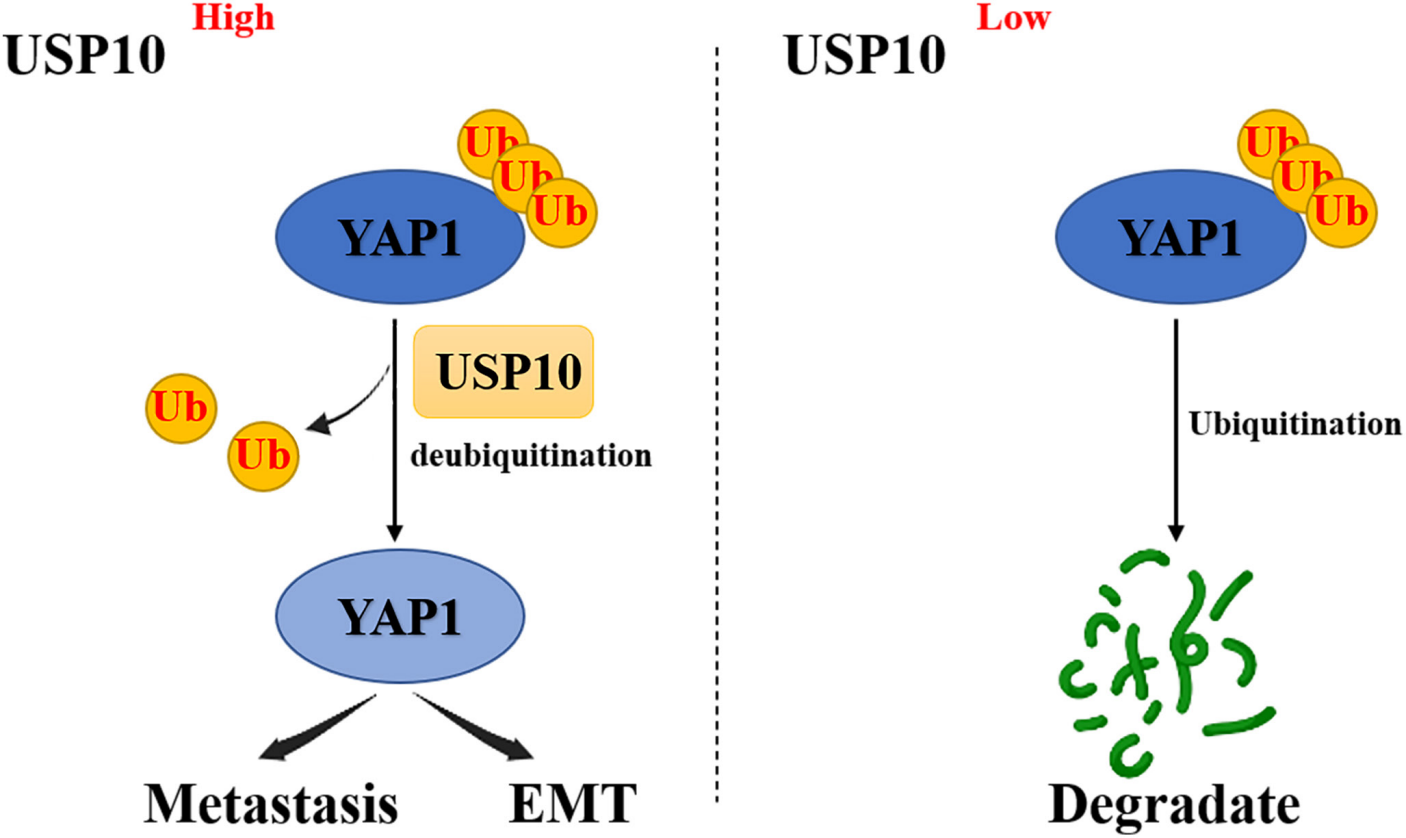
Proposed model for the mechanism through which USP10 promotes the metastasis and epithelial‐mesenchymal transition of osteosarcoma (OS) cells by regulating the expression of YAP1.

Based on these results, we can conclude that USP 10 changes the degradation and ubiquitin of YAP1 in osteosarcoma cells, thus stabilizing the expression of YAP1 (Figure 9).

## 4 DISCUSSION

Despite rapid developments in medicine, including in the fields of surgery, radiotherapy, and chemotherapy, the 5‐year survival rate of patients with OS is low, with one of the reasons being the metastasis of OS in the early stages. Therefore, understanding the mechanism of OS metastasis can provide new avenues for OS treatment. The metastasis of malignant tumors is a complicated process that is affected by many factors. Research has noted that EMT performs a crucial part in distant metastasis,^17^, ^32^ including breast,^33^ liver,^34^ prostate cancers,^35^ and OS cells.^36^ Studies have found that EMT and distant metastasis in OS is strongly correlated with the abnormal expression of oncogenes in tumor cells.^37^ This study proved that increased USP10 expression is a predictor of poor OS prognosis and has a pivotal contribution in EMT and metastasis in OS.

USP10 is a member of the deubiquitinating enzyme family.^10^ Its molecular functional structures are mainly cysteine‐type endopeptidase and ubiquitin sulfhydryl esterase. Yuan et al. found that, USP10 mainly localizes in the cytoplasm and regulates p53 homeostasis. Following DNA damage, USP10 also translocates to the nucleus and contributes to p53 activation.^38^ Recent studies have shown that USP10 plays different roles in different tumors. Lin et al. showed that USP10 inhibits the occurrence and development of colon cancer by deubiquitinating SIRT6.^39^ Sun et al. found that USP10 inhibits the proliferation and metastasis of tumor cells by upregulating PTEN expression.^16^ Takayama et al. found that USP10 can inhibit P53 by deubiquitinating G3BP2.^12^ Yuan et al. found that USP10 can deubiquitinate TGF‐β to promote distant metastasis of hepatocellular carcinoma in Smad4‐positive patients.^13^ These investigations have demonstrated that USP10 has a key contribution to malignant tumors. Our study findings revealed that the expression of USP10 in cancerous tissues was increased compared with that in the paracancerous tissues of patients with OS. The expression of USP10 was correlated with tumor size, distant metastasis, and TNM stage in OS patients. Further in vivo and in vitro experiments in this work have shown that high USP10 expression can promote EMT and metastasis in OS cell lines. In conclusion, our experiments indicate that USP10 may affect OS progression as an oncogene and affect OS progression.

EMT induces a variety of signaling pathways, such as the Hippo‐YAP signaling pathway.^40^ Prior research has revealed that the Hippo/YAP signaling pathway is unusually active in multiple cancers and is widely included in their biological processes,^41^ including liver cancer,^42^ pancreatic cancer,^43^ and glioma.^44^ Therefore, to elucidate the pathway through which USP10 controls EMT, we decided to focus on the Hippo‐YAP signaling pathway's YAP1 downstream protein. First, we discovered that USP10 and YAP1 expressions elevated in OS cell lines and cancerous tissues and that the two were positively correlated. On the one hand, silencing USP10 in OS cell lines downregulated YAP1 expression and simultaneously reduced the EMT ability, resulting in a decrease in distant metastasis of OS. On the other hand, USP10 overexpression promoted YAP1 expression, improved EMT, and simultaneously increased distant tumor metastasis. Upregulation of YAP1 can reverse the decrease in EMT caused by USP10 silencing and increase the distant metastasis of OS cells, while downregulation of YAP1 can weaken the increase in EMT caused by USP10 overexpression and decrease the distant metastasis of OS cells. In summary, these results indicate that USP10 affects EMT and metastasis in OS by regulating the expression of YAP1.

Research has noted that USP10 can influence the biological functions of cells by limiting ubiquitination and the breakdown of substrate proteins. In our previous study,^26^ we found that YAP1 was degraded by ubiquitination. Interestingly, in this study, it was found to USP10 affects the protein levels of YAP1 but not its mRNA levels. Therefore, we speculated that USP10 might stabilize YAP1 protein levels by deubiquitinating it. Moreover, through co‐IP experiments, Zhu et al. found that USP10 directly binds to YAP1 in liver cancer cells.^30^ In this study, we found that USP10 can directly bind to YAP1 in OS cell lines. Reduce ubiquitinated YAP1 by deubiquitination, thereby preventing its degradation by the ubiquitin‐protease system and stabilizing the expression of YAP1.

## 5 CONCLUSION

In conclusion, our findings indicate that USP10 and YAP1 expressions increased in OS tissues and cell lines. Our study also showed that USP10 is associated with disease progression and poor prognosis in osteosarcoma patients. Simultaneously, we also observed that USP10 could affect the EMT and osteosarcoma cells' invasiveness and migration in vivo and in vitro. Through co‐IP, we found that USP10 can directly interact with YAP1 to reduce ubiquitinated YAP1, thereby stabilizing its protein levels and affecting EMT and distant metastasis in OS cells. Therefore, this study indicated that USP10 is a potential predictive factor for diagnosing EMT and distant metastasis in OS and is a possible future treatment.

## AUTHOR CONTRIBUTIONS

**Jianyong Deng:** Conceptualization (lead); data curation (lead); formal analysis (lead); validation (lead); writing – original draft (lead). **Xuan Yi:** Conceptualization (equal); formal analysis (equal); methodology (equal); validation (equal); writing – original draft (equal). **Zuxi Feng:** Investigation (equal); methodology (equal). **Jie Peng:** Validation (equal). **Dan Li:** Methodology (equal); validation (equal). **Chen Li:** Supervision (equal). **Binbin Deng:** Formal analysis (equal). **Shuaigang Liu:** Formal analysis (equal). **Souradeep Sahu:** Validation (equal). **Liang Hao:** Conceptualization (equal); funding acquisition (lead); supervision (lead); writing – review and editing (lead).

## FUNDING INFORMATION

This study was supported by grants from the National Natural Science Foundation of China (Nos. 81760487 and 82060492), College Students' Innovative Entrepreneurial Training Plan Program (No. 202110403003), and Project of the Jiangxi Provincial Department of Science and Technology (No. 20212ACB216011).

## CONFLICT OF INTEREST STATEMENT

No potential conflict of interest was reported by the author(s).

## ETHICS STATEMENT

The Ethics Committee of the Second Affiliated Hospital of Nanchang University approved the use of human data and tissues. Each participant signed a written informed consent form prior to the study. All animal experiments were approved by the Animal Experimental Ethics Committee of the Second Affiliated Hospital of Nanchang University.

## Data Availability

The datasets generated and analyzed during the current study are available from the corresponding author upon reasonable request.
